# Study on Preparation of Polymer-Modified Bentonite and Sand Mixtures Based on Osmotic Pressure Principle

**DOI:** 10.3390/ma15103643

**Published:** 2022-05-19

**Authors:** Chunyang Zhang, Xi Wei, Chaocan Zhang, Yinchun Li, Yitian Sheng, Shu Peng

**Affiliations:** School of Materials Science and Engineering, Wuhan University of Technology, Wuhan 430070, China; 13476089628@163.com (C.Z.); 303868@whut.edu.cn (X.W.); liyinchun@whut.edu.cn (Y.L.); chrysemy@163.com (Y.S.); ps15771189488@163.com (S.P.)

**Keywords:** bentonite, polymer-modified, swell index, hydraulic conductivity, osmotic pressure, self-healing

## Abstract

Polymer-modified bentonite and sand mixtures (PMBS) are widely used in the engineering field due to their low cost and low permeability. In this study, different ionic types of polyacrylamides were used to modify bentonite to improve its swelling properties and impermeability. The physicochemical properties of polymer-modified bentonite were characterized by X-ray diffraction, particle size distribution, IR spectroscopy, SEM, and free swell index (FSI) to further demonstrate the successful organic modification of bentonite. To investigate the impermeability mechanism of PMBS from the perspective of osmotic pressure, the colloidal osmotic pressure of bentonite and hydraulic conductivity were compared. The results showed that anionic polyacrylamide (APAM) had the most obvious improvement on the swelling properties of bentonite, and 3% APAM increased the FSI of bentonite from 15 mL/2 g to 41 mL/2 g. With the increase in polymer dosage, the colloidal osmotic pressure of bentonite increased and the hydraulic conductivity of PMBS decreased significantly. The interior of PMBS is equivalent to a highly concentrated bentonite–sand–water system. When the colloidal osmotic pressure in the restricted space is higher than the external hydraulic pressure, it will prevent infiltration from occurring. When the external hydraulic pressure exceeds the high concentration of bentonite colloid osmotic pressure, the hydraulic conductivity may increase rapidly. Therefore, the impermeability of PMBS depends on the colloidal osmotic pressure of bentonite. Finally, it was confirmed that PMBS had a self-healing capacity by simulating damage to PMBS.

## 1. Introduction

Municipal solid waste has become a significant environmental problem as a result of population growth, economic development, and urbanization [1]. In 2018, China’s effective waste disposal capacity expanded to 226 million tons per year, with sanitary landfills accounting for 52 percent of the total volume [2]. However, the decomposition of household garbage in landfills generates hazardous effluent that endangers the soil and groundwater surrounding some landfills [3,4]. Therefore, it makes sense to design an effective artificial barrier system in the face of the complex challenge of increasing waste generation [5].

Bentonite is a natural clay formed primarily of the montmorillonite family, consisting of two silica tetrahedral lamellae and one alumina octahedron lamellae [6,7]. It is frequently utilized in environment-related materials due to its excellent swelling properties and low permeability [8,9,10]. To protect soil and groundwater from pollution, landfills often use compacted clay liners (CCL), geosynthetic clay liners (GCL), geomembranes, and bentonite–sand mixtures (BS) as barrier systems [11,12]. However, CCL often cracks because of uneven settlement or dryness, which significantly weakens its impermeability. GCL and geomembranes may also lose impermeability due to punctures from sharp stones or roots [13,14]. Some studies have shown that BS with 20% bentonite content meets the minimum standards for landfills, which usually have a hydraulic conductivity of less than 10^−9^ m/s [15,16]. However, with global environmental awareness growing, common bentonite can hardly meet the increasing environmental standards and engineering needs. Therefore, scholars conduct a lot of polymer modification research on bentonite, which greatly improves its swelling and impermeability properties [17]. Not only that, polymers have the ability to chelate and adsorb, which facilitates the dispersion of bentonite and increases its specific surface area, enabling it to adsorb heavy metal ions and organic pollutants. Finally, polymer-modified bentonite is mixed with sand to prepare PMBS impermeable materials with excellent performance and very low permeability [18,19,20].

Studies on polymer-modified bentonite (PMB) and its impermeability mechanism have been an important research hotspot in the fields of materials and the environment. Theng [21] proposed that the interaction between clay and polymer can be classified based on the ionic charge on the polymer surface. Negatively charged polymers tend to be repelled by the net negative charge of the clay particles and stick to the surface, whereas positively charged polymers bind to the internal negative charge of the clay by electrostatic forces. Haase and Schan [22] used the cluster model to theoretically analyze the effect of polymer adsorption on the variation of hydraulic conductivity, which may be strongly related to the PMB pore size distribution. The adsorption of cationic and anionic polymers promoted the formation of aggregated and flocculated clay fabrics, respectively, while nonionic polymers promoted the formation of dispersed clay fabrics. Geng [23] suggested that the lower permeability of polymer-treated GCL can be attributed to the formation of hydrogels due to the adsorption of water molecules onto the polymer chains, and this strong interaction leads to an increase in the shear strength of the permeable fluid. Guler [12] concluded that the hydraulic conductivity decreases with increasing intrinsic permeability due to the increased viscosity of the polymer-treated soil mixture. Yu [24] confirmed that the permeability of the material is reduced because the tiny cavity structure of PMB causes many obstacles when the liquid flows through the matrix. There are also many researchers who believe that clay materials exhibit semi-permeable membrane behavior, referring to the ability of clay to restrict the migration of dissolved chemicals or solutes [25,26,27]. The impermeability mechanism of PMB is very complex, and almost no researchers have explored the process of permeability from the perspective of material osmotic pressure. We believe that the impermeability of PMBS is determined by the colloidal osmotic pressure of bentonite, so this paper will analyze the impermeability behavior of PMBS based on the principle of osmotic pressure.

In actual projects, the barrier system may be damaged by uneven ground surfaces, plant roots, etc. The self-healing capacity of GCL maintains its low hydraulic conductivity. Sari and Chai [28] found that GCL’s self-healing capacity is primarily determined by the swelling properties and thickness of bentonite. Yu [29] showed that the size of the damage hole has a significant impact on the self-healing capacity of GCL. When the damage hole diameter was small (2 mm), GCL was easy to self-heal. However, when the damaged hole reached approximately 15% of the specimen area, the specimen lost its self-healing capacity. There are still few studies on the self-healing capacity of PMBS, which also has excellent self-healing capacity due to the high swelling properties of PMB.

At present, there is no research to explore the impermeability mechanism of polymer-modified bentonite from the perspective of material osmotic pressure. The main objective of this work is to investigate the impermeability of PMBS based on the principle of osmotic pressure. For this purpose, we used the solution method to organically modify bentonite and mixed it with sand in proportion to prepare PMBS impermeable material. For the first time, a theoretical model of the PMBS impermeability mechanism was proposed from the perspective of osmotic pressure. The relationship between the impermeability of PMBS and the osmotic pressure was investigated by testing the colloidal osmotic pressure of bentonite and hydraulic conductivity. The self-healing capability of PMBS was verified by simulating the damage caused by stones and plant roots. The physicochemical properties of PMB were characterized by XRD, PSD, IR, and SEM. The research results can provide new ideas and theoretical support for the application of polymer-modified bentonite in the field of impermeability.

## 2. Materials and Methods

### 2.1. Materials

Natural sodium bentonite (BT) was provided by Jilin Liufangzi bentonite technology company. It is a gray-white powder with a moisture content of 10%, a particle size of 75 μm, a blue absorption of 31.67 g/100 g, a montmorillonite content of 71.65%, a cation exchange capacity of 70.48 mmol/100 g, and a free swell index of 15 mL/2 g. The general structural formula of bentonite is (1/2Ca, Na)_0.66_(Al, Mg, Fe)_4_[Si_4_Al_8_O_20_](OH)_4_·nH_2_O, and its basic chemical composition is shown in Table 1. It was used after being dried in an oven at 105 °C for 24 h. The sand was dried, ground, and then used after passing through a 30-mesh standard sieve. Anionic polyacrylamide (APAM), cationic polyacrylamide (CPAM), and nonionic polyacrylamide (NPAM) were all industrial-level products produced in Wuxi, Jiangsu. The water used during the experiments was deionized.

### 2.2. Preparation of Polymer-Modified Bentonite

Polymer-modified bentonite (PMB) was prepared as follows: Firstly, 30 g of BT was added to 500 mL of water and stirred for 2 h to make it fully dispersed in the water. Different amounts of polymers (APAM, CPAM, NPAM) were dissolved in 100 mL of water at 80 °C. Secondly, the polymer solution was added to the bentonite dispersion and stirred fully in the mixer for 2 h. Then, the PMB slurry was removed and aged at room temperature for 1 day. Finally, the PMB slurry was put into an oven at 105 °C for 48 h, and after the sample was dried, it was ground to 200 mesh. The polymer dosage was 1%, 2%, 3%, 4%, and 5% of the relative bentonite mass, respectively.

### 2.3. Preparation of Polymer-Modified Bentonite and Sand Mixtures

**Figure 1:**
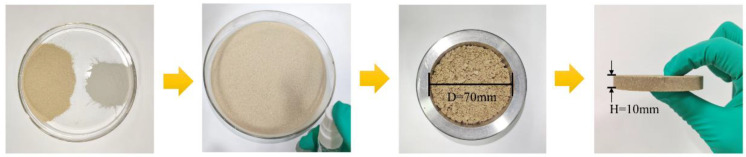
Schematic diagram of PMBS preparation.

According to the above experimental results, we found that APAM-modified bentonite had the best swelling properties, and thus it was mixed with sand to prepare PMBS specimens. As shown in Figure 1, PMB*x* (*x* means the added amount of APAM) and sand were mixed in a certain ratio for a total of 70 g. PMB*x* accounted for 10%, 15%, and 20% of the total mass, respectively. Then, we used a small spray bottle to add 10 g of water to the mixed sample, stirring while spraying. After mixing well, we put it into a mold and pressed it into a round cake with a diameter of 70 mm and a height of 10 mm.

### 2.4. Material Characterization

X-ray diffraction (XRD) was performed using a Panaco Empyrean diffractometer from Almelo, Netherlands, radiating the samples with Cu (λ = 1.5406 Å) at 40 kV and 40 mA. Scans were recorded between 4° and 35° with a step size of 0.05° and a scan speed of 2°/min. Particle size distribution (PSD) measurements of bentonite colloids (1 g/L) were performed using the Malvern Mastersizer 2000 laser particle size analyzer from England, which can measure a wide range of sizes with high accuracy. FTIR was used to test the changes of modified bentonite using a Nexus intelligent Fourier transform infrared spectrometer from Therno Nicolet, USA, with a measured spectral range of 400–4000 cm^−1^ and an accuracy of 0.01 cm^−1^, 64 scans were recorded with a resolution of 4 cm^−1^. Scanning electron microscope (SEM) was used to characterize the microscopic morphology of BT, PMB, and PMBS using the JSM-7500F from Tokyo, Japan.

### 2.5. Free Swell Index

The impermeability of PMBS was strongly associated with the swelling behavior of bentonite. Therefore, free swell test was performed on bentonite according to the American Society for Testing and Materials’ method ASTM D5890. Firstly, the 200-mesh bentonite sample was dried to a constant weight. Then, add 90 mL of water to a 100 mL measuring cylinder. Then, 2 g of bentonite was added to the cylinder in batches, slowly adding 0.1 g of sample each time, with an interval of not less than 10 min between two adjacent times. Finally, water was added to 100 mL in the cylinder, and the free swell index of bentonite (mL/2 g) was measured after 24 h.

### 2.6. Hydraulic Conductivity Tests

**Figure 2:**
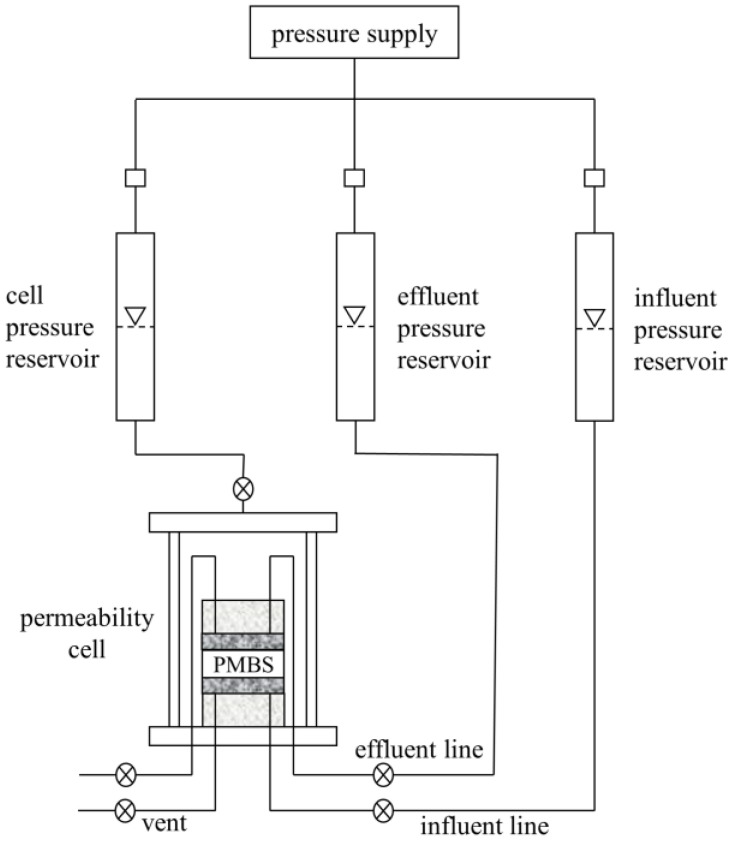
Diagram of the hydraulic conductivity test configuration.

Hydraulic conductivity is the most direct index to judge the impermeability of the material. In this paper, the hydraulic conductivity was tested using a flexible wall permeameter with reference to the method of ASTM D5084. As shown in Figure 2, the permeation cell base was first installed with permeable stone, filter paper, the PMBS specimen, filter paper, and permeable stone from bottom to top, and then the flexible membrane was placed around the specimen and fixed with “O” rings. In the first stage, after filling the permeability cell with water, the specimen was given a surrounding pressure of 35 kPa and initial backpressure of 15 kPa above and below, and this state was maintained for 48 h so that the specimen reached saturation. In the second stage, the influent pressure was adjusted to the designated pressure so that the water penetrated the specimen from the bottom up. Finally, the hydraulic conductivity of PMBS was measured at different hydraulic pressure, and the hydraulic conductivity k can be calculated by Equation (1).

(1) $$k=\frac{a\cdot L}{2\cdot A\cdot ∆t}\ln\left(\frac{∆h_{1}}{∆h_{2}}\right)$$

where *a* is the area of the reservoirs containing either the influent or effluent liquid (m^2^); *L* is the height of the specimen (m); *A* is cross-sectional area of specimen (m^2^); Δ*t* is the time interval (s); Δ*h*_1_ is the head loss across the permeameter/specimen at the start time of the permeation trial (m); Δ*h*_2_ is the head loss across the permeameter/specimen at the end time of the permeation trial (m).

### 2.7. Osmotic Pressure Tests

To verify the osmotic pressure effect of PMBS, different masses of BT and PMB were dispersed in water, and after stirring, sonication, and shaking, bentonite dispersions of different mass concentrations were prepared. Then, the osmotic molar concentrations of bentonite colloids were measured by a German GONOTEC freezing point osmotic pressure meter, Osmomat030 3000. Finally, osmotic pressure was calculated by the simple osmotic pressure equation π = (n/V) RT proposed by Van’t Hoff, where n/V is the molar concentration of solute in solution [30].

### 2.8. Self-Healing Capacity

During the actual construction process, PMBS may be defective due to uneven ground and plant roots, which may affect its impermeability performance. The PMB has excellent swelling properties, so some of the defects may achieve self-healing and the impermeability performance is restored as before. Therefore, this paper simulated the damage caused by plant roots and stones to PMBS to explore the self-healing capacity. Firstly, a 2 mm diameter hole and a 10 mm diameter hemispherical hole were drilled in two complete PMBS specimens, respectively. Then, the specimens were transferred to the permeameter and pre-saturated for 48 h. Finally, the change in hydraulic conductivity of the damaged specimens over 120 h was measured at 15 kPa hydraulic pressure.

## 3. Results and Discussion

### 3.1. Material Characterization

#### 3.1.1. X-ray Diffraction (XRD)

**Figure 3:**
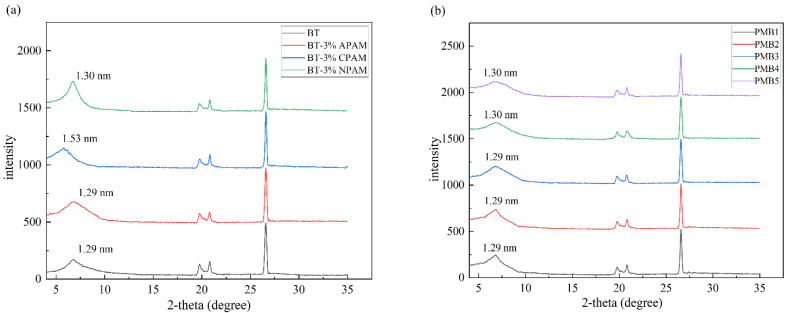
(**a**) XRD patterns of different polymer-modified bentonites, (**b**) XRD patterns of PMB with different content of APAM.

Figure 3a shows the XRD patterns of the different polymer-modified bentonites. It can be seen that the characteristic reflective surface crystal spacing *d*_001_ of BT is 1.29 nm (2θ = 6.868°), which is typical of sodium-based bentonite [31]. After modification with 3% APAM and NPAM, the layer spacing is 1.29 nm and 1.30 nm, respectively, without significant changes. Additionally, after 3% CPAM modification, the layer spacing increases from 1.29 nm to 1.53 nm, which has a significant intercalation effect. This is due to the presence of the net negative charge between the bentonite layers, which attracts the cationic chain segments in CPAM and causes them to intercalate between the bentonite layers. While the negatively charged APAM is repelled by the net negative charge of the bentonite and attracted by the positively charged edge bentonite particles, which are bridged to the bentonite surface by hydrogen bonds or van der Waals forces. The bonding between NPAM and bentonite is likely to take place mainly through ion-dipole interactions/coordination and hydrogen bonding [32,33]. Figure 3b shows the XRD patterns of PMB with different content of APAM. It can be seen that with the increase in APAM content, the characteristic peaks of montmorillonite gradually become smooth. This may be due to the introduction of COO- hydrophilic groups, which leads to a decrease in the crystallinity of montmorillonite. As a result, PMB has improved water absorption and swelling properties.

#### 3.1.2. Particle Size Distribution (PSD)

**Figure 4:**
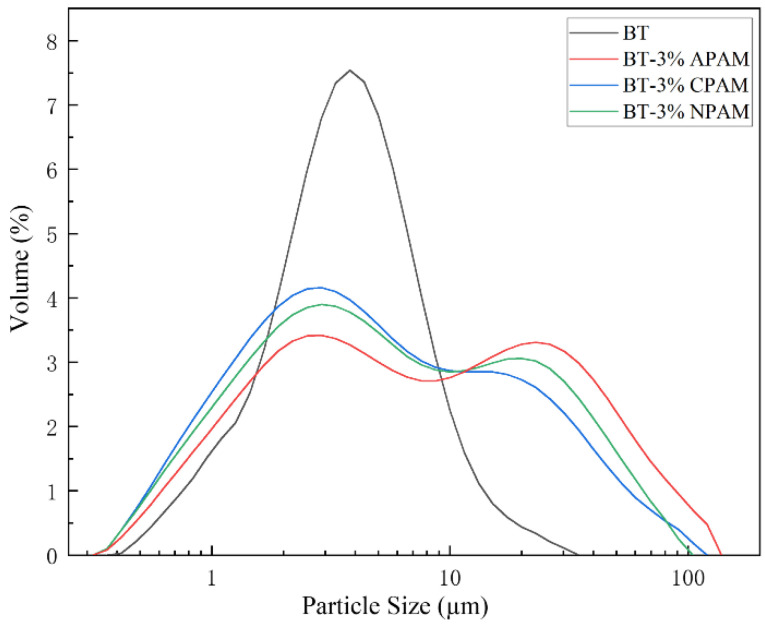
Particle size distribution of different polymer-modified bentonite colloids.

Figure 4 shows the particle size distribution of different polymer-modified bentonite colloids. It can be seen that the unmodified bentonite shows a normal distribution peak, while the PMB shows two normal distribution peaks and shifts to the right side of the size distribution curve. The volume average particle size of BT is 4.542 μm. The volume average particle size of BT is 4.542 μm. After 3% APAM, CPAM, and NPAM modifications, the particle size increases to 11.955 μm, 8.175 μm, and 9.184 μm, respectively. PSD and swelling properties are closely related, so polyacrylamide can better enhance the swelling properties of bentonite, and APAM-modified bentonite has the best effect.

#### 3.1.3. Infrared Spectroscopy (IR)

**Figure 5:**
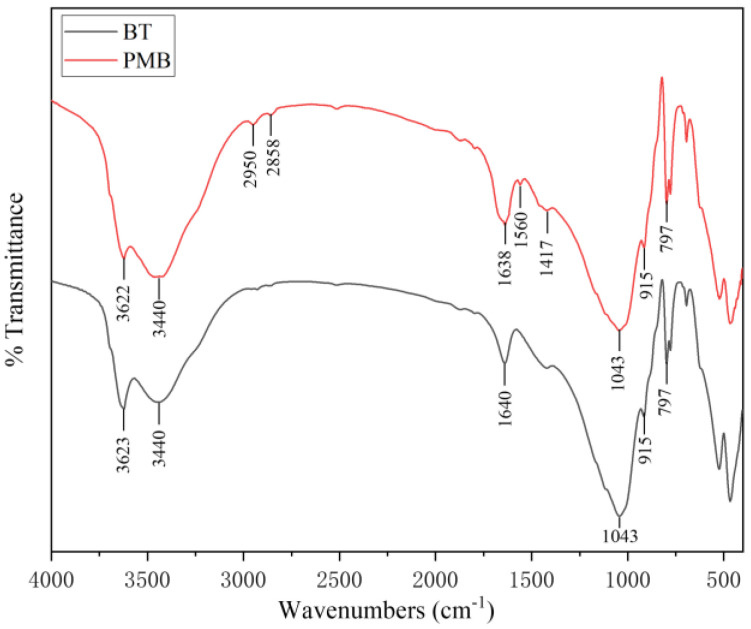
Comparison of the infrared absorption spectroscopy of BT and PMB.

Figure 5 shows the infrared spectroscopy of BT and APAM-modified bentonite. The main spectral bands of bentonite are: 3623 cm^−1^ attributed to the stretching vibration of montmorillonite structural hydroxyl -OH, 3440 cm^−1,^ and 1640 cm^−1^ mainly due to the stretching vibration of an interlayer water molecule -OH and bending vibration of -OH, 1043 cm^−1^ and 797 cm^−1^ attributed to the stretching vibration and bending vibration of Si-O, and 915 cm^−1^ attributed to the bending vibration of montmorillonite structural hydroxyl -OH [8,34]. After APAM modification, the peak around 3440 cm^−1^ becomes broader, which may be caused by the asymmetric stretching vibration of -NH_2_. The new peaks at 2950 cm^−1^ and 2858 cm^−1^ are stretching vibration peaks of -CH_3_ and -CH_2_, respectively [35,36]. The absorption band intensity increases at 1638 cm^−1^, which is related to the stretching vibration of C=O and the bending vibration of -NH_2_. The 1560 cm^−1^ is due to the acrylate COO- and 1417 cm^−1^ is due to the C-N stretching vibration [32]. Therefore, it can be seen that PMB does not change the structure of the original bentonite, further confirming that APAM mainly modifies the bentonite surface.

#### 3.1.4. SEM

**Figure 6:**
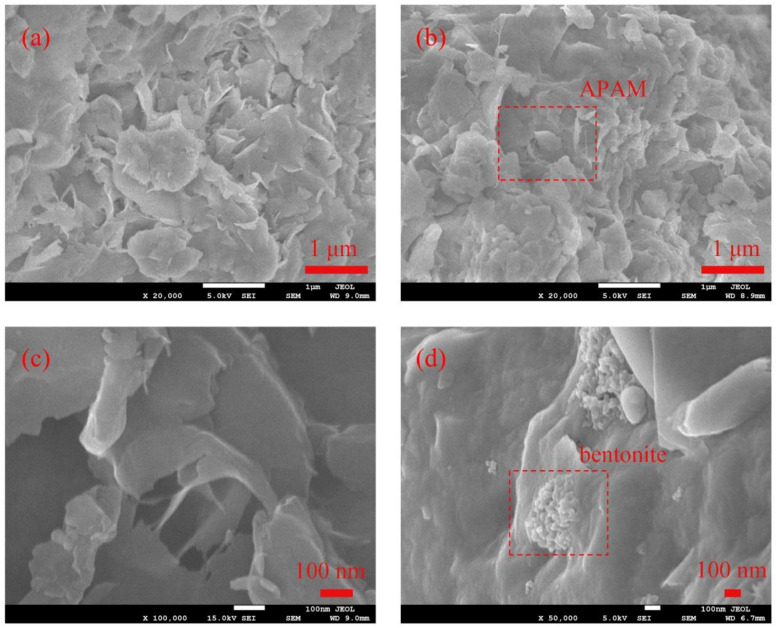
(**a**) SEM images of BT, (**b**) and (**c**) SEM images of PMB, (**d**) SEM image of PMBS.

Figure 6 shows the SEM images of BT, PMB, and PMBS. As can be seen from Figure 6a, BT is a flat, layer-by-layer stacked sheet structure [37]. Figure 6b,c shows that the surface of the bentonite modified by APAM has changed significantly. The bentonite lamellae become irregular, while the polymeric mesh structure appears at the edges of the bentonite lamellae. APAM is connected between different lamellae due to the repulsion of negative charges between the bentonite layers. This is further evidence that APAM successfully modified the bentonite surface [22,38]. Figure 6d is an SEM image of the internal section of PMBS, which shows that the PMB and sand are stacked together completely and closely, and the sand provides a restricted space for the PMB. Therefore, when external water infiltrates, the osmotic pressure potential is generated within the restricted space to prevent infiltration from occurring.

### 3.2. Free Swell Index

**Figure 7:**
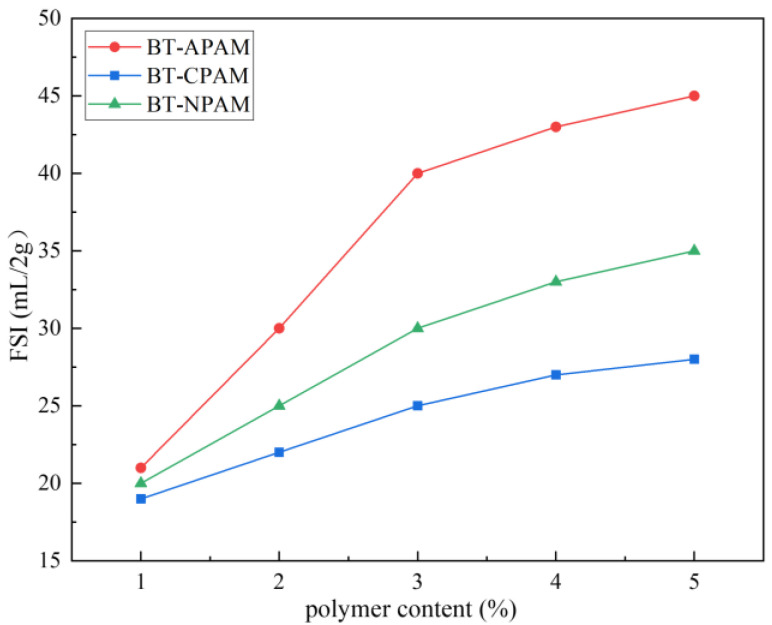
The free swell index of PMB with different polymer content.

In order to select a suitable polymer modifier, the free swell index (FSI) of PMB with different content of APAM, CPAM, and NPAM was measured separately. It can be seen from Figure 7 that the FSI of PMB gradually increases with the increase in polymer content, and the swelling properties are improved more obviously at the modifier dosage of 3%. When the polymer content is 3%, the FSI of BT modified by APAM, CPAM, and NPAM increases to 41 mL/2 g, 25 mL/2 g, and 30 mL/2 g, and the swelling properties improve by 173%, 66%, and 100%, respectively. The FSI test results are highly consistent with the PSD results. This is due to the interaction between polyacrylamide and bentonite in water, which makes the bentonite more hydrophilic. More water molecules flow into the interlayer of bentonite, which increases the thickness of the diffusion bilayer and improves the swelling properties [39,40]. CPAM is mainly intercalated between bentonite layers by the interaction force of negative charges between cations and bentonite layers, which may form agglomerates due to the electrostatic force and affect the swelling properties. APAM and NPAM are mainly bridged to the bentonite surface by hydrogen bonds or van der Waals forces and interact with the bentonite to improve the swelling properties [38,41]. However, APAM-modified bentonite has anions present on the surface, so it has better dispersion in water, resulting in higher swelling properties. The impermeability of PMBS strongly depends on the swelling properties of bentonite, so we chose APAM as the bentonite modifier.

### 3.3. Osmotic Pressure and Hydraulic Conductivity

**Figure 8:**
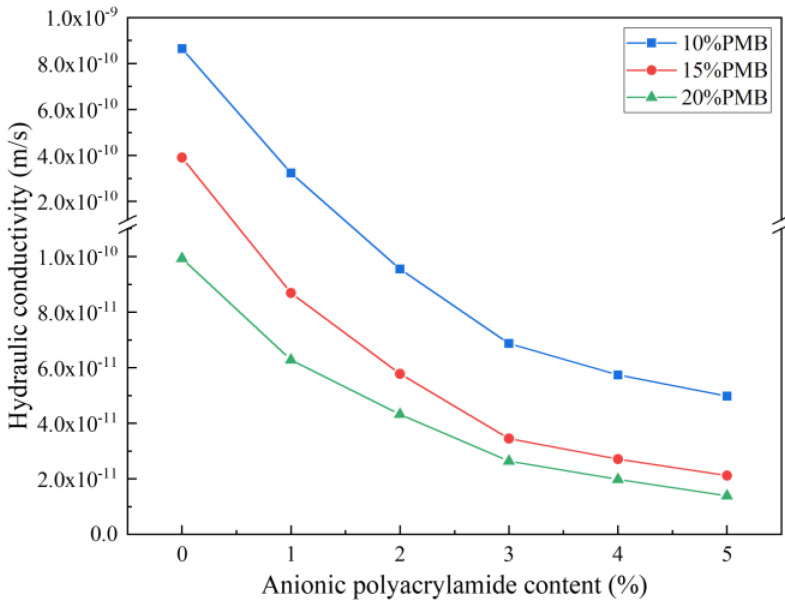
Change of hydraulic conductivity with respect to APAM content.

**Figure 9:**
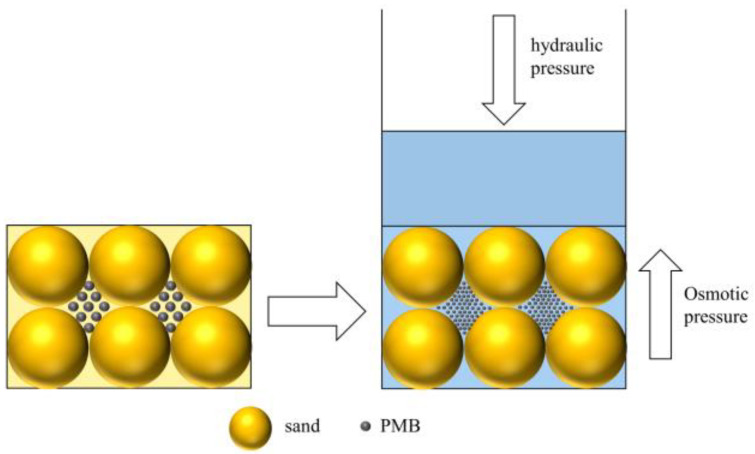
Schematic diagram of PMBS impermeability principle.

According to the previous research results, the hydraulic conductivity of the bentonite–sand composite system is usually about 10^−9^–10^−10^ m/s. Akgun [42] evaluated the permeability properties of bentonite in southern Turkey and measured that the hydraulic conductivity of the bentonite–sand mixtures was 8.73 × 10^−10^ m/s. Demdoum [43] conducted hydraulic conductivity tests on composites with 10% bentonite, 20% sand, and 70% tuff, and the result was 1.44 × 10^−^^10^ m/s. The addition of polymer significantly reduces the hydraulic conductivity of bentonite–sand mixtures. Figure 8 shows the relationship between the change of PMBS hydraulic conductivity and the content of APAM at different bentonite–sand ratios when the hydraulic pressure is 15 kPa. It can be seen that the hydraulic conductivity of PMBS gradually decreases with the increase in APAM content. When bentonite accounts for 15%, the hydraulic conductivity of BS is 3.91 × 10^−10^ m/s. After 3% APAM modification, the hydraulic conductivity of PMBS is reduced to 3.45 × 10^−11^ m/s, which is a whole order of magnitude lower. With the further increase in APAM content, there is a slight decrease in hydraulic conductivity, and after 5% APAM modification, the hydraulic conductivity of PMBS is reduced to 2.12 × 10^−11^ m/s. This trend also exists when bentonite accounts for 10% and 20% of the total mass. However, when the amount of PMB is increased from 10% to 15%, the impermeability of PMBS is improved more obviously, and the sand provides a more suitable restricted space for bentonite. If the content of PMB is too high, it may lead to high plasticity and the mixture will be difficult to compact [44]. As a result of cost and property considerations, the optimal amount of PMB is 15%. We suggest that the decrease in hydraulic conductivity may be an osmotic pressure effect. As shown in Figure 9, for the compacted PMBS, the bentonite and sand are tightly packed together. Sand is equivalent to many rigid spheres, providing restricted space for the bentonite, so little bentonite is lost. When external water starts to infiltrate, the bentonite forms a highly concentrated colloid with a relatively high osmotic pressure. According to the theory of thermodynamics, a system with high osmotic pressure will spontaneously reduce the osmotic pressure due to water absorption. However, in the restricted space of high-density PMBS, it is difficult for highly concentrated bentonite colloids to absorb water and swell. It means that the external water cannot get in and lower the osmotic pressure, thus acting as an impermeability. If the hydraulic pressure is higher than the internal osmotic pressure of PMBS, the osmotic balance will be destroyed, and external water will forcibly infiltrate, resulting in a decrease in impermeability. Polymer addition makes the colloidal osmotic pressure of bentonite increase, so PMBS has lower hydraulic conductivity.

**Figure 10:**
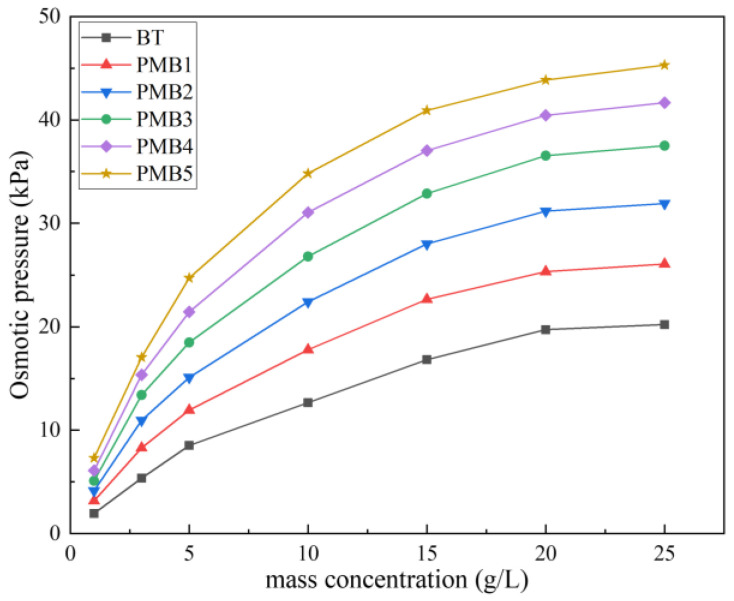
Osmotic pressure of bentonite colloids with different mass concentrations.

**Figure 11:**
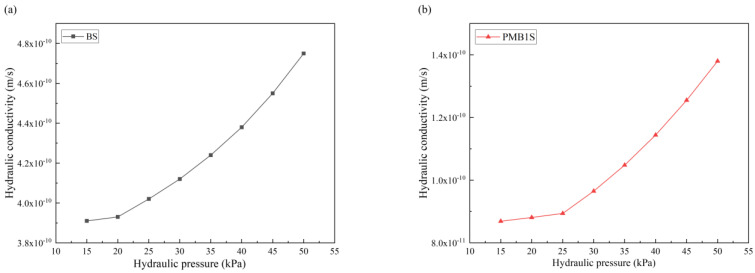

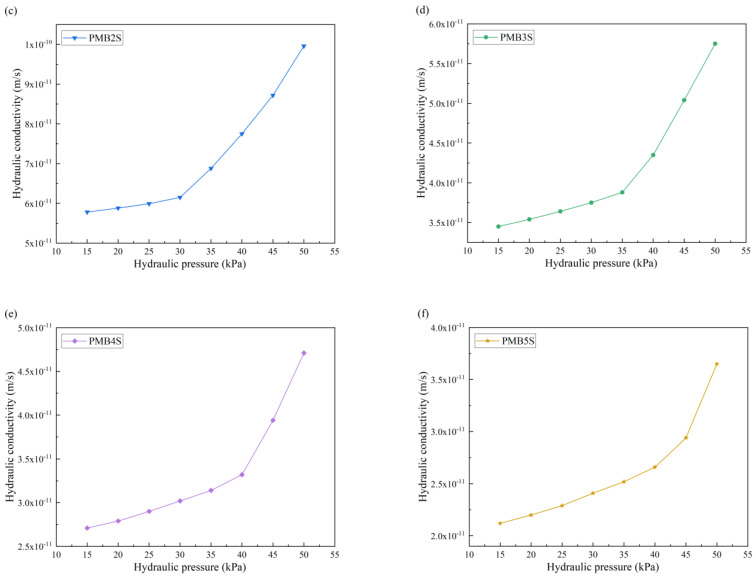
Change in hydraulic conductivity of BS and PMBS under different hydraulic pressure (**a**) BS, (**b**) PMB1S, (**c**) PMB2S, (**d**) PMB3S, (**e**) PMB4S, (**f**) PMB5S.

To further confirm the osmotic pressure effect of PMBS, osmotic pressure tests were performed on BT and PMB colloids of different mass concentrations, respectively. Then the changes in hydraulic conductivity of BS and PMBS under different hydraulic pressures were tested. As shown in Figure 10 and Figure 11, the higher the content of APAM, the higher the colloidal osmotic pressure of PMB, and the better the impermeability of PMBS. When the high mass concentration state is reached, the increase in osmotic pressure gradually tends to level off, which may be due to the presence of some suspended particles in the bentonite colloid not playing a role. In the high mass concentration state, the osmotic pressure of unmodified BT is 20.21 kPa, while the osmotic pressure of PMB3 with 3% APAM increases to 36.54 kPa. As shown in Figure 11a, when the hydraulic pressure is less than 20.21 kPa, the change in hydraulic conductivity of BS is minor, whereas when the hydraulic pressure exceeds 20.21 kPa, the hydraulic conductivity appears to increase significantly and rapidly. Its hydraulic conductivity increases to 4.77 × 10^−10^ m/s at 50 kPa hydraulic pressure. As shown in Figure 11d, when the hydraulic pressure is lower than 36.54 kPa, the hydraulic conductivity of PMB3S is basically unchanged, while when the hydraulic pressure exceeds 36.54 kPa, the hydraulic conductivity starts to increase gradually. Its hydraulic conductivity reaches 5.75 × 10^−11^ m/s at 50 kPa hydraulic pressure. This trend of hydraulic conductivity variation is also present in PMB1S, PMB2S, PMB4S, and PMB5S. For compacted BS or PMBS, the interior is equivalent to a high concentration of bentonite–sand–water system. Therefore, when the osmotic pressure in the restricted space is higher than the external hydraulic pressure, it will be better for impermeability. However, since the ideal osmotic pressure effect cannot be achieved at high concentrations, bentonite appears to be suspended, so there may be a threshold value. When the external hydraulic pressure exceeds this threshold, the hydraulic conductivity may increase rapidly.

### 3.4. Self-Healing Capacity

**Figure 12:**
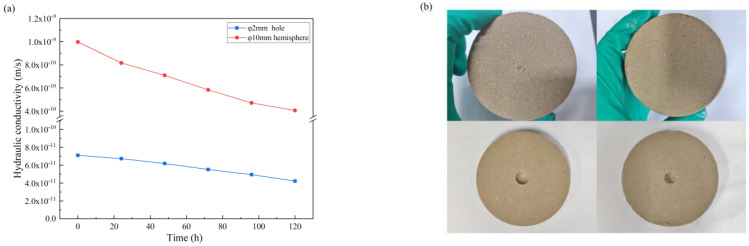
(**a**) The relationship between hydraulic conductivity and time for PMBS with different damage degrees, (**b**) the change of PMBS with different damage degrees over 120 h.

Since PMB has excellent swell properties, PMBS has a certain self-healing capacity. As shown in Figure 12a, the hydraulic conductivity of PMBS with a 2 mm diameter hole is 7.10 × 10^−11^ m/s at the beginning of the permeation test, which is equivalent to twice that of the intact PMBS specimen. With the passage of time, the damaged specimens gradually undergo repair, and the hydraulic conductivity gradually decreases. The hydraulic conductivity decreases to 4.22 × 10^−11^ m/s after 120 h, which is close to that of the intact PMBS specimens. In addition, the starting hydraulic conductivity of PMBS with a 10 mm diameter hemispherical hole is 9.97 × 10^−10^ m/s, which is nearly 30 times larger than that of the intact PMBS specimen. Despite the extent of damage, the hydraulic conductivity recovers to 4.06 × 10^−10^ m/s after 120 h, which is only an order of magnitude higher than the intact PMBS specimens. Figure 12b shows that after 120 h, the PMBS with a 2 mm diameter hole basically completes self-healing and the hole almost disappears, and the PMBS with a 10 mm diameter hemispherical hole also achieves partial self-healing. Because of the high swelling properties of PMB, when external water penetrates the PMBS specimen, the internal swelling pressure pushes the bentonite–sand to fill the holes to achieve self-healing [45]. In actual projects, PMBS is generally laid 70 mm–100 mm thick, so even in the face of defects caused by uneven ground, plant roots, etc., PMBS can almost achieve self-healing without affecting its impermeability.

## 4. Conclusions

In this study, the PMB with high swelling properties was prepared by modifying natural sodium-based bentonite with different ionic types of polyacrylamides. The FSI and PSD measurements show that the APAM-modified bentonite has the highest swelling properties. The XRD results show that CPAM is attracted by electrostatic forces to intercalate between the bentonite layers, while APAM and NPAM are mainly bridged to the bentonite surface by hydrogen bonds or van der Waals forces. The IR and SEM results are further evidence of the successful modification of the bentonite surface by APAM. When PMB accounts for 15%, the sand provides a more suitable restricted space for bentonite. At 15 kPa hydraulic pressure, the hydraulic conductivity of PMBS with 3% APAM is 3.45 × 10^−11^ m/s, which is a full order of magnitude lower than that of BS. Even at 50 kPa hydraulic pressure, its hydraulic conductivity still reaches 5.75 × 10^−11^ m/s. This work proposed the osmotic pressure effect of PMBS that the higher the osmotic pressure of bentonite, the better the impermeability performance. For compacted PMBS, the interior is equivalent to a high concentration of bentonite–sand–water system. When the osmotic pressure in the restricted space is higher than the external hydraulic pressure, it will prevent infiltration from occurring. However, since the ideal osmotic pressure effect cannot be achieved at high concentrations, there may be a threshold value. When the external hydraulic pressure exceeds this threshold, the hydraulic conductivity may increase rapidly. Finally, it is confirmed that PMBS has some self-healing capacity by simulating the damage caused by stones and plant roots to PMBS. Therefore, PMBS, which is a low-cost, low-permeability, self-healing artificial barrier system, has a wide range of application prospects in landfill and industrial impermeability fields.

In this paper, a theoretical model of the impermeability mechanism of PMBS under ideal conditions is proposed. In fact, there may be partially connected voids between the sands inside the PMBS. Under long-term hydraulic pressure, some bentonite colloids may absorb water and swell to reduce the osmotic pressure, thus affecting impermeability. Therefore, the long-term impermeability of PMBS can be further evaluated by permeation tests. In addition, the regularity between hydraulic conductivity and osmotic pressure of PMBS can be further explored by using waste filtrate as permeate fluid.

## Figures and Tables

**Table 1:** Chemical composition of natural sodium bentonite (% mass).

SiO_2_	Al_2_O_3_	Fe_2_O_3_	MgO	Na_2_O	CaO	K_2_O	LOI
64.2	15.05	2.97	1.80	2.66	1.60	0.92	7.38

## Data Availability

The data presented in this study are available on request from the corresponding author.
